# Commercial Scale Manufacturing of Allogeneic Cell Therapy

**DOI:** 10.3389/fmed.2018.00233

**Published:** 2018-08-22

**Authors:** Gary M. Pigeau, Elizabeth Csaszar, Aaron Dulgar-Tulloch

**Affiliations:** ^1^Centre for Advanced Therapeutic Cell Technologies, Toronto, ON, Canada; ^2^General Electric Healthcare, Cell Therapy Technologies, Marlborough, MA, United States; ^3^Centre for Commercialization of Regenerative Medicine, Toronto, ON, Canada

**Keywords:** allogeneic, pluripotent stem cell, stirred-tank reactor, bioreactor, cell therapy, manufacturing, scale-up, regenerative medicine

## Abstract

Allogeneic cell therapy products are generating encouraging clinical and pre-clinical results. Pluripotent stem cell (PSC) derived therapies, in particular, have substantial momentum and the potential to serve as treatments for a wide range of indications. Many of these therapies are also expected to have large market sizes and require cell doses of ≥10^9^ cells. As therapeutic technologies mature, it is essential for the cell manufacturing industry to correspondingly develop to adequately support commercial scale production. To that end, there is much that can be learned and adapted from traditional manufacturing fields. In this review, we highlight key areas of allogeneic cell therapy manufacturing, identify current gaps, and discuss strategies for integrating new solutions. It is anticipated that cell therapy scale-up manufacturing solutions will need to generate batches of up to 2,000 L in single-use disposable formats, which constrains selection of currently available upstream hardware. Suitable downstream hardware is even more limited as processing solutions from the biopharmaceutical field are often not compatible with the unique requirements of cell therapy products. The advancement of therapeutic cell manufacturing processes to date has largely been developed with a cell biology driven approach, which is essential in early development. However, for truly robust and standardized production in a maturing field, a highly controlled manufacturing engineering strategy must be employed, with the implementation of automation, process monitoring and control to increase batch consistency and efficiency.

## Introduction

The field of cell therapy is maturing both scientifically and commercially, with over 600 cell therapy clinical trials being reported (https://alliancerm.org/page/arm-q3-2017-quarterly-data-report). Although the majority of the recent publicized cell therapy developments have been focused on autologous or patient-matched immune-oncology products, there continues to be steady progress in the advancement of allogeneic cell therapeutics. As these therapies progress toward clinical application, establishing robust commercial scale manufacturing solutions will be essential.

The most mature allogeneic cell therapies are mesenchymal stem cell (MSC) or fibroblast derived, which are being investigated both for their lineage differentiation potential and their immunomodulation and paracrine signaling effects (1). At present, a small number of approved MSC-based products exist in limited jurisdictions, including Prochymal and TEMCELL for the treatment of graft vs. host disease (GvHD) and CARTISTEM for osteoarthritis. Specific considerations for scaling up MSC production have been reviewed elsewhere (2). The immunotherapy field, while primarily pursuing autologous models at present, has key industrial players, such as Cellectis and Celyad, who are developing allogeneic CAR-T and related products, and it remains to be seen how much of an impact allogeneic approaches will ultimately have. Pluripotent stem cell (PSC) derived therapies are currently in earlier development; however, early clinical trial successes have been demonstrated, particularly in the area of retinal pigment epithelium (RPE) for the treatment of macular degeneration (3), neural lineage cells for the treatment of spinal cord injury (4), and pancreatic beta cells for the treatment of insulin-dependent diabetes (5). Companies with candidate products in or nearing clinical trials in these areas include: Astellas and Healios for RPEs; Asterias for PSC-derived oligodendrocyte progenitor cells; Viacyte and Semma Therapeutics for pancreatic beta cells. A comprehensive list of PSC-derived cell therapies in clinical trials has recently been published (6). To date, PSC-derived cell products being evaluated in the clinic are typically being cultured at small scales using traditional manual tissue culture approaches. Pre-clinical development in PSC-derived cardiomyocytes, neurons, and hepatocytes is also emerging, with the potential of serving large markets. As therapies mature and larger markets are targeted, developing scalable manufacturing solutions has become a priority focus for the field. Although there are key manufacturing differences between products of different lineages, many of the commercial scale manufacturing considerations are consistent.

Cell dosing requirements for these PSC therapies remain uncertain, but estimates put dosing needs at up to 10^9^ cells/patient. With market sizes for some therapies anticipated to be in the tens-to-hundreds of thousands of doses, it is expected that commercial scale manufacturing will need to accommodate production of 10^11^-10^14^ cells per year for a single product. The cell therapy field is just exploring how to handle batch production for these cell numbers. It is expected that commercial allogeneic scale batch sizes will end up being between 200 and 2,000 L to facilitate high dose requirements while balancing considerations of cost and operational efficiency, process stability, cell doubling considerations, and risk of batch losses.

Large-scale manufacturing is standardized and well-controlled in the bioprocessing field and cell therapy is positioned to adopt many of these solutions. There are, however, several considerations that make cell therapy manufacturing unique. These include requirements to carefully maintain or control cell identity and potency, the need to recover functional cells at the end of culture as opposed to soluble components, and the inability to sterile filter the final drug product. Currently, cell therapy manufacturing remains largely driven by a cell biology mindset. This has been essential to establish mechanism of action and demonstrate the production of high quality cells in early development. As the field matures however, it will be critical to apply a manufacturing engineering mindset to these processes. In the following sections, we outline current solutions and forward-looking recommendations to advance cell therapy manufacturing toward commercial scale production, with a focus on PSC-derived therapies.

## Scale-up hardware

To manufacture the anticipated 10^11^-10^14^ annual cells/indication requirements, scale-up of the traditional plastic-adherent PSC culture workflows presents significant space, labor, logistical and cost challenges, as demonstrated for MSC production (7). In contrast, a suspension-based approach, preferably without the need for microcarriers to avoid their increased cost, workflow and particulate disadvantages, provides a scalable manufacturing strategy. To achieve 200–2,000 L batch sizes, single-use stirred tank reactors (STRs) likely remain the preferred hardware for scale-up due to their long history of success in bioprocess production, excellent process control, flexibility in start-up and change over, and well-characterized transfer kinetics between reactor scales. There is an increasing body of work within our lab and others describing PSC expansion in aggregate suspension culture, demonstrating these cells are amenable to growth in STRs without the need for microcarriers, in a manner which offers cost efficient scale-up and downstream purification advantages (8, 9). Operationally, single-use disposable bioreactors are mostly identical to their stainless-steel counterparts, with control and monitoring of process parameters key to production of the therapeutic product (pH, dissolved gases via aeration, temperature, agitation and media provision regimes). However, single-use STRs have several advantages. Their increased operational expense is offset by efficiencies in vessel preparation, turnaround, and line change-over, as well as removal of steam sterilization and chemical cleaning requirements, and a reduction in both batch- and cross-contamination risk. GE Healthcare (GEHC; Xcellerex), Pall (Allegro), and Sartorius (BIOSTAT) offer manufacturing platforms of single-use bioreactors with designs that closely match traditional bioprocessing STRs. The scales available in these product lines are amenable to large scale allogeneic manufacturing and provide a progressive scale of bioreactors which would facilitate scale-up seed train design (Table 1).

**Table 1:** Comparison of single-use disposable stirred tank reactor platforms.

**Reactor**	**Min volume (mL)**	**Max volume (mL)**	**Turndown ratio**	**Aspect ratio**	**Scale-up factor**	**21 CFR 11 compliance**	Single use available^**^	**Agitation**
**STIRRED TANK REACTOR SYSTEMS SCALING TO** ≤ 10 L^*^								
**Applikon**								
MiniBio 250	50	200	4.0	1.6:1	–	Yes	No	Direct drive, lip sealed, Rushton or marine impellers
MiniBio 500	100	400	4.0	1.5:1	2.0	Yes	No	Direct drive, lip sealed, Rushton or marine impellers
MiniBio 1000	200	800	4.0	1.9:1	2.0	Yes	No	Direct drive, lip sealed, Rushton or marine impellers
Standard-1	300	900	3.0	2.3:1	–	Yes	No	Direct drive, lip sealed, Rushton or marine impellers
Standard-2	500	1,700	3.4	2.3:2	1.9	Yes	No	Direct drive, lip sealed, Rushton or marine impellers
Standard-3	500	2,700	5.4	2.3:3	1.6	Yes	Yes−3 L	Direct drive, lip sealed, Rushton or marine impellers
**Eppendorf**								
DASBox	60	250	4.2	4.0:1	–	Yes	Yes−0.38 L	Overhead drive, marine, Rushton or pitched blade
DASGip-2.5	750	2,700	3.6	3.1:1	–	Yes	Yes−1.8 L	Overhead drive, pitched blade
DASGip-3.5	850	3,800	4.5	3.5:1	1.4	Yes	Yes−5 L	Overhead drive, pitched blade
BioFlo 120-1	400	1,000	2.5	2.7:1	–	Yes	Yes−1.8 L	Direct or magnetic, Rushton, pitched blade, marine or spin filter
BioFlo 120-2	800	2,200	2.8	2.7:1	2.2	Yes	Yes−1.8 L	Direct or magnetic, Rushton, pitched blade, marine or spin filter
BioFlo 120-5	2,000	5,600	2.8	2.0:1	2.5	Yes	Yes−5 L	Direct or magnetic, Rushton, pitched blade, marine or spin filter
BioFlo 120-10	4,000	10,500	2.6	2.3:1	1.9	Yes	Yes−14 L	Direct or magnetic, Rushton, pitched blade, marine or spin filter
BioFlo 320-1	600	1,900	3.2	2.6:1	–	Yes	Yes−1.8 L	Direct or Magnetic, Rushton, pitched blade, or marine, spin filter, cell lift or packed-bed
BioFlo 320-3	1,300	3,800	2.9	2.5:1	2.0	Yes	Yes−1.8 L	Direct or Magnetic, Rushton, pitched blade, or marine, spin filter, cell lift or packed-bed
BioFlo 320-5	1,900	5,600	2.9	2.4:1	1.5	Yes	Yes−5 L	Direct or Magnetic, Rushton, pitched blade, or marine, spin filter, cell lift or packed-bed
BioFlo 320-10	3,500	10,500	3.0	2.3:1	1.9	Yes	Yes−14 L	Direct or Magnetic, Rushton, pitched blade, or marine, spin filter, cell lift or packed-bed
**Finesse**								
G3 Lab-1	500	1,000	2.0	1.9:1	–	Yes	No, but controller compatible with major single use platforms	Direct drive, Rushton, pitched blade, marine
G3 Lab-3	1,200	2,000	1.7	1.9:1	2.0	Yes		Direct drive, Rushton, pitched blade, marine
G3 Lab-7	2,500	5,000	2.0	2.3:1	2.5	Yes		Direct drive, Rushton, pitched blade, marine
G3 Lab-15	5,000	10,000	2.0	NA	2.0	Yes		Direct drive, Rushton, pitched blade, marine
**Infors**								
Multifors	75	250	3.3	NA	–	Yes	No, but controller compatible with major single use platforms	Magnetic drive, Rushton or pitched blade
	150	500	3.3	NA	2.0	Yes		Magnetic drive, Rushton or pitched blade
	220	750	3.4	NA	1.5	Yes		Magnetic drive, Rushton or pitched blade
MiniFors	NA	1,500	NA	NA	–	Yes	No, but controller compatible with major single use platforms	Direct drive, Rushton, pitched blade
	NA	3,000	NA	NA	2.0	Yes		Direct drive, Rushton, pitched blade
	NA	6,000	NA	NA	2.0	Yes		Direct drive, Rushton, pitched blade
Labfors	500	1,200	2.4	NA	–	Yes	No, but controller compatible with major single use platforms	Magnetic drive, Rushton or pitched blade
	500	2,300	4.6	NA	1.9	Yes		Magnetic drive, Rushton or pitched blade
	1,000	5,000	5.0	NA	2.2	Yes		Magnetic drive, Rushton or pitched blade
	2,100	7,000	3.3	NA	1.4	Yes		Magnetic drive, Rushton or pitched blade
	2,200	10,000	4.5	NA	1.4	Yes		Magnetic drive, Rushton or pitched blade
**Sartorius**								
BIOSTAT A/B-1	400	1,000	2.5	1.6:1	–	NA	No	Direct drive, 3 blade segment impeller
BIOSTAT A/B-2	600	2,000	3.3	1.8:1	2.0	NA	Yes−2 L	Direct drive, 3 blade segment impeller
BIOSTAT A/B-5	600	5,000	8.3	2.2:1	2.5	NA	No	Direct drive, 3 blade segment impeller
BIOSTAT A/B-10	1,500	10,000	6.7	2.5:1	2.0	NA	No	Direct drive, 3 blade segment impeller
**Reactor**	**Min volume (L)**	**Max volume (L)**	**Turndown ratio**	**Aspect ratio**	**Scale-up factor**	**21 CFR 11 compliance**	**Single use available**	**Agitation**
**STIRRED TANK REACTOR SYSTEMS SCALING TO 2,000 L**								
**GE Healthcare**								
XDR-10	4.5	10	2.2	1.5:1	–	Yes	Yes	Bottom-mount, magnetic drive, 3 blade pitched
XDR-50	22	50	2.3	1.5:1	5.0	Yes	Yes	Bottom-mount, magnetic drive, 3 blade pitched
XDR-200	40	200	5	1.5:1	4.0	Yes	Yes	Bottom-mount, magnetic drive, 3 blade pitched
XDR-500	100	500	5	1.5:1	2.5	Yes	Yes	Bottom-mount, magnetic drive, 3 blade pitched
XDR-1000	200	1,000	5	1.5:1	2.0	Yes	Yes	Bottom-mount, magnetic drive, 3 blade pitched
XDR-2000	400	2,000	5	1.5:1	2.0	Yes	Yes	Bottom-mount, magnetic drive, 4 blade pitched
**Pall**								
Allegro 200	60	200	3.3	1:01	–	Yes	Yes	Bottom-mount, direct drive, 3 blade “elephant ear”
Allegro 1000	300	1,000	3.3	1:01	5.0	Yes	Yes	Bottom-mount, direct drive, 3 blade “elephant ear”
Allegro 2000	400	2,000	5	1:01	2.0	Yes	Yes	Bottom-mount, direct drive, 3 blade “elephant ear”
**Sartorius**								
BIOSTAT STR 50	12.5	50	4	1.8:1	–	In development	Yes	Top-mount, magnetic drive, mechanical seal, dual impeller, 3 or 6 blade pitched
BIOSTAT STR 200	50	200	4	1.8:1	4.0	In development	Yes	Top-mount, magnetic drive, mechanical seal, dual impeller, 3 or 6 blade pitched
BIOSTAT STR 500	125	500	4	1.8:1	2.5	In development	Yes	Top-mount, magnetic drive, mechanical seal, dual impeller, 3 or 6 blade pitched
BIOSTAT STR 1000	250	1,000	4	1.8:1	2.0	In development	Yes	Top-mount, magnetic drive, mechanical seal, dual impeller, 3 or 6 blade pitched
BIOSTAT STR 2000	500	2,000	4	1.8:1	2.0	In development	Yes	Top-mount, magnetic drive, mechanical seal, dual impeller, 3 or 6 blade pitched

* *Some vendors have systems >10 L*.

** *For ≤ 10 L systems, specifications are for standard sterilizable glass vessels. Single use vessels are based whether the vendor manufactures an option*.

*These are volumetrically matched and may differ slightly in configuration from glass vessels*.

*NA, specification not available at vendor's website*.

In this comparison, we focus on single-use bioreactor platforms with a scalable path to 2,000 L. Although we have not comprehensively reviewed smaller (Eppendorf, Applikon, Infors, Finesse, Sartorius, etc.) or alternate format platforms (rocking bed, rotating wheel, vibrating disk, oscillating, orbital, etc.), these may be utilized to generate the cell mass for inoculation of the larger manufacturing bioreactors. A survey of small stirred-tank reactors, capable of scaling from ≤1 to ≤10 L is provided in the first section of Table 1. While there are a variety of vendors and configurations available, the Eppendorf platforms (BioFlo 120 and 320) offer single-use vessels which span across available scales. However, some platforms are also compatible with off-brand single-use vessels. Growth kinetics and maximal cell densities will influence the volumetric scale-up steps required and the availability of single-use, current good manufacturing practice (cGMP) compliant vessels requires consideration for PSC seed train development. The requirements of the smaller format platforms will vary with cell type and have been reviewed with respect to PSC production separately (10). For commercial scale production, the Xcellerex product line offers the lowest volumetric barrier to entry with the XDR-10 and its minimum working volume of 4.5 L, followed by the BIOSTAT 50 at 12.5 L and the Allegro 200 at 60 L. Assuming a constant inoculation target across the three platforms, the BIOSTAT and Allegro platforms would require 3- and 13-fold greater initial cell mass, respectively, to initiate a seed train to scale to the 2,000 L manufacturing volume.

As the process scales up, the turndown ratio of the next largest reactor becomes important as the final produced cell mass from the preceding reactor must be sufficient to maintain appropriate inoculation levels with a minimum of seed train steps. Assuming a consistent inoculation and output cell concentration across each platform, leveraging the turndown ratio by inoculating at the minimum volume and fed-batch feeding to its maximum volume would be required in all the second stage reactors. That said, with differing turndown ratios comes differing fed-batch strategies. Assuming turndown ratios in the range of 1:2–1:5 and cell doubling times on the order of 24 h, filling times at constant cell density would range from roughly 48–72 h. This leads to significant differences in feed rates, with approximately 1, 4, 20, and 35 L/h feed rates for 50, 200, 1,000, and 2,000 L reactors, respectively. For a given process or cell line, this may impact the choice of platform as high flow rates may affect the bioreactor environment and cell culture conditions.

Both GEHC and Sartorius maintain traditional vessel geometries, with aspect (h/d) ratios >1:1 (Table 1). Pall provides an alternate geometry of 1:1, which allows for operational advantages with respect to access to the vessel at height, especially at the largest scales, but does increase the manufacturing footprint. For process development activities, availability of small scale reactors with comparable vessel geometries and fluid dynamic properties is an important consideration. In our lab we utilize the Eppendorf 200 mL (DASbox, h/d = 1.5) and 1 L (BioFlo 320, h/d = 1.4) platforms to generate inoculum for the XDR-10 (h/d = 1.5). While we have seen adequate translation of process parameters between these platforms, differences in vessel configuration and impeller design of the XDR-10 have necessitated optimization of agitation rate to minimize shear and recapitulate growth kinetics established in the smaller scale, process development STR platforms.

Agitation and impeller options differ across the platforms. An in-depth consideration of available STR platforms is available (11). Of the platforms discussed here, pitched blade impellers are available from GEHC and Sartorius. These configurations have 3–6 flat blades at 35–45° angles, and offer both radial and axial mixing. Pall offers an elephant ear configuration and this is a segmented 3-blade which provides axial mixing. Both impellor offerings provide gentle mixing of mammalian cell cultures. The choice of impeller may be influenced by several factors including shear sensitivity of the cell line and k_L_A requirements. Due to differences in fluid flow, a pitched blade impeller offers increased mass transfer at lower agitation rates compared to an elephant ear configuration. The Sartorius platform offers a unique dual-impeller configuration which would conceptually allow for more homogeneous mixing throughout the vessel's working volume. However, if a fed-batch process were to begin at a volume lower than the upper impeller, the culture would experience increased turbulence, possibly resulting in increased hydrodynamic stress and foaming, as the working volume crossed over the impeller threshold.

Significant gaps in the currently available technology include an integrated perfusion solution and media formats. Perfusion requires an additional unit operation [such as tangential flow filtration (TFF) or alternating tangential flow (ATF)] to enable media replenishment and waste removal while retaining cells in the bioreactor. With low growth rates and relatively high perfusion rates required, traditional chemostat operation of the bioreactor is not possible. In order to avoid issues of hydrodynamic and shear stress, STRs are typically run with headspace sparging and low agitation rates, which result in oxygen mass transfer limitations in culture that impact achievable culture densities. Other modifications to these reactor systems may also be required for therapeutic cell production, such as improved consumable quality/bag welds to ensure sterility is maintained, changes in materials of construction or extractables and leachables to account for more sensitive primary cells, or changes in port design and placement to allow for in process monitoring of parameters relevant for therapeutic cells.

In addition to proposed reactor modifications, commercial scale cell therapy production will necessitate other adaptations from tool and regent suppliers. For example, provision of media to the large-scale single-use bioreactor poses a limitation to scale up. The complex, costly media currently required for therapeutic cells are typically available in 500 mL−1 L format and are manufactured under small lot conditions. Manufacturers of allogeneic cell therapy media will need to plan for larger format batches and end-use packaging. A move to powdered formulations and onsite preparation will require a production facility to invest in water for injection (WFI) and may comprise a contamination risk and therefore a critical control point in the overall process. Identifying mimetic-based alternatives to costly growth factors or leaner media alternatives would also help to substantially reduce cost of goods. Culture media and additives are currently a major cost driver for allogeneic cell therapy manufacturing and incremental improvements to achieve cost reduction will have substantial impact at commercial scale.

## Process definition, monitoring, control, and batch records

As allogeneic manufacturing approaches the maturity of traditional biological manufacturing, many of the process monitoring and control approaches from that field will be adopted and refined to reflect the stringent requirements for process definition and control. Fully automated process controls will be implemented and continuous process data will define tolerable operational ranges. As the industry matures to industrialized production from process development and a focus on biological understanding, sampling of the process will be limited to those required to inform process intervention decisions.

An example of where process automation can enable the removal of discrete sampling is perfusion feed rate cascade control and viable cell counts. Bioreactor culture of PSCs and the need for perfusion-based feeding is complicated by the fact that slow growth rates and need for rapid media replenishment necessitates a cell retention device. Despite decades of establishment of this specific feedback cascade control (12), there have been no reports of automating perfusion feed rates in response to available PSC bioreactor process data. Dissolved oxygen (DO) and pH of the cell culture media are typically held constant to provide a consistent environment to support optimal cell expansion. Both DO and pH probes produce a process signal in response to increased cellular need for oxygen and a lactic acid dependent decrease in pH. Work in our lab has demonstrated that these signals, alone or in combination, can be used to provide a cascade control of the media feed pump and produce proxy measures for viable cell density. This aspect of process automation can be used to remove sampling requirements and operator input on run conditions, thereby producing a more consistent, metabolically driven control scheme.

**Figure 1:**
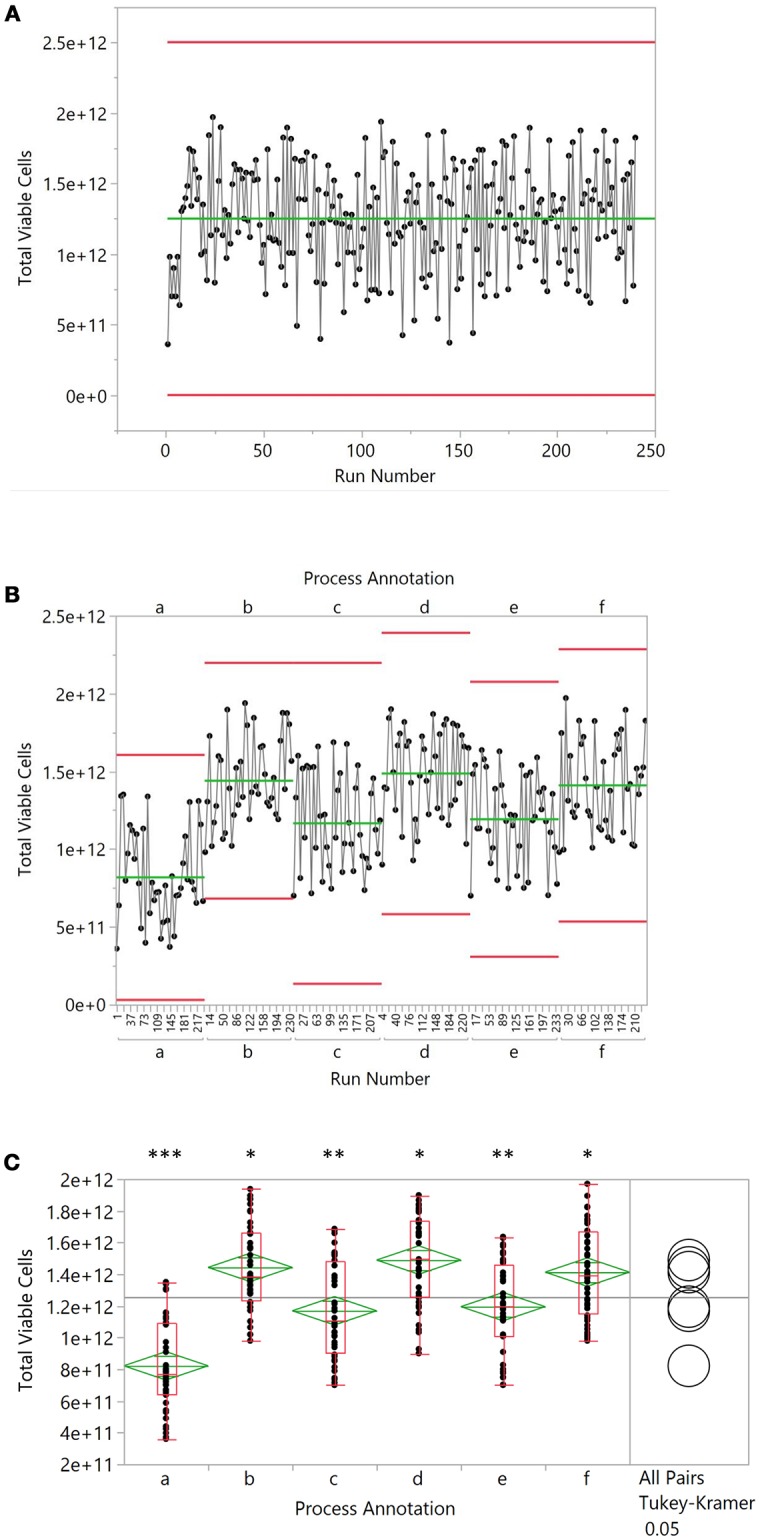
Simulated production data from 240 batches. An average cell number of 1.3 × 10^12^ was chosen as a starting point. Excel-based, random number generation was used to simulate variability in the data, resulting in a coefficient of variation of 27%. Batch data was numbered sequentially and every 20 batches, the mean was adjusted up or downward by 3–6 × 10^12^ cells to create six discrete groupings labeled “a” through “f.” Groups were then ordered alphabetically and by batch number to yield the randomized data in **(A)**. When grouped by alphabetic grouping, the data was organized as shown in **(B)**. Lastly, groupings were subjected to an ANOVA analysis with a Tukey-Kramer *post-hoc* test (*p* < 0.05), where a different number of asterisks indicate significantly different means **(C)**.

Xbar and r charts have a long history in process definition, statistical process control and continuous improvement of manufacturing processes (13) and the central principles remain relevant to cell therapy manufacturing. As processes become more defined, and an understanding of the contributing factors of product variation becomes developed, the industry will need to transition to early detection and prevention of out of specification processes and ultimately, products. Variation may be the result of a number of known and manageable factors, including manufacturing crew, rotating seed train vessels, growth factor lots, or production vessels in a multi-line facility. However, when simple process run outputs are viewed in sequence, this variation often appears to be unexplainable and is often assumed to have a stochastic biological cause. By employing process annotation, the true cause of process differences can emerge. Figure 1 illustrates this in a simplified manner using simulated data from a scaled-up allogeneic manufacturing scenario. In this example, a run history is presented (Figure 1A) with a process that appears to be subject to biological variation. However, the importance of process annotation is drawn out in Figure 1B where the data has been re-grouped according to a specific process variable and a pattern emerges. While overall variance within each group is roughly equivalent, they demonstrate a significant difference in performance when described relative to each other (Figure 1C). The goal for this sort of analysis is a decrease in the number of unknown causes of variation and identification of known causes. Continuous process improvement, Six Sigma and Lean manufacturing can then be applied to increase process reliability and product quality.

## Downstream processing

Downstream processing of cell therapy products is likely the most significant area of divergence from the traditional bioprocessing space. While upstream cultivation of cell therapy products can be performed in hardware originally designed for bacterial or CHO cell production with relatively minor hardware adaptation, many of the needs for cell harvest, wash, and formulation are very different. Most critically for cell therapeutics, the cells themselves are the drug product and must be recovered with consistently high viability and maintenance of quality and potency. Conversely, hardware employed in the protein and viral production fields are designed to retain the drug product in the culture media and leads to the cells being destroyed, damaged, or non-recoverable.

Additionally, the biological requirements of the culture can create further complexities, such as the need to disaggregate traditionally adherent cells prior to harvest. Typically, enzymes are used for dissociating aggregates in the culture vessels as available harvesting solutions typically require cells to be reduced to a single cell suspension prior to harvest. This introduces challenges around enzyme addition, mixing, appropriate disruption post-incubation, and enzyme removal or inactivation within a short period of time to prevent cell damage. A disruptive technology in the field would either enable rapid non-enzymatic cell disaggregation, or provide a solution whereby aggregates could be harvested and formulated without disaggregation.

At present, there are limited hardware solutions to accommodate downstream needs of cell therapies in a closed and scalable manner. Options include counter-flow centrifugation, such as the KSep (Sartorius) and the Elutra cell separation system (Terumo BCT), continuous centrifugation, and TFF. There have been minimal demonstrations of downstream processing of PSCs or their differentiated progeny using a scalable solution, and as such it is difficult to assess suitability of the current hardware on cell recovery and quality. One challenge is these devices are often not available in scaled-down versions, which can limit process development and suitability demonstrations. To accommodate the varied needs of downstream processing of cell therapy products, novel separation solutions may need to be developed, potentially by adapting technologies from other fields.

Once cultured cells have been harvested and formulated, they must undergo final filling. Cell therapy products will likely be filled in cryovials or cryobags to enable cryopreservation and improved storage and shipping logistics. There are currently several vendors marketing vial filling hardware. These are marketed toward the generation of master cell banks (MCBs) and working cell banks (WCBs), but the same systems are suitable for commercial scale filling of cell therapies. Options available include vendors who are marketing liquid handlers to fill third party cryo-compatible vials (Hamilton, TAP/Sartorius, VanRX, AST) and vendors aiming to provide an integrated solution of vials, filling device, cryostorage solutions and thawing (COOK Regentec, Aseptic Tech, MedInstill). In either case, these tools have not yet been proven at the envisioned commercial scale, and challenges exist around consistency between first and last fill and exposure time to potentially detrimental cryoprotectants. Furthermore, filling solutions for bag-based packaging are extremely scarce.

## Summary

The cell therapy industry is developing quickly and the current FDA approvals of the autologous immuno-oncology products Kymriah (Novartis) and Yescarta (Gilead) may signify an inflection point in the field. As allogeneic therapies mature, creating a mature manufacturing industry will be essential. Where suitable, adapting strategies and hardware from bioprocessing and other manufacturing industries will accelerate progress in cell therapy. In some instances, gaps remain that will necessitate new technology development. As cell therapy manufacturing emerges, technology developers will play a critical role in ensuring the hardware and integration solutions keep up with clinical developments.

## Author contributions

All authors listed have made a substantial, direct and intellectual contribution to the work, and approved it for publication.

### Conflict of interest statement

GP and AD-T are employed by GE Healthcare, Cell Therapy Technologies. The Xcellerex bioreactor platform is a GE Bioprocess product. The remaining author declares that the research was conducted in the absence of any commercial or financial relationships that could be construed as a potential conflict of interest.
